# Perspective: Stepping Stones to Unraveling the Pathophysiology of Mal de Debarquement Syndrome with Neuroimaging

**DOI:** 10.3389/fneur.2018.00042

**Published:** 2018-02-12

**Authors:** Viviana Mucci, Yoon-Hee Cha, Floris L. Wuyts, Angelique Van Ombergen

**Affiliations:** ^1^Antwerp University Research Centre for Equilibrium and Aerospace, Department of Biomedical Physics, Faculty of Sciences, University of Antwerp, Antwerp, Belgium; ^2^Laureate Institute for Brain Research, Tulsa, OK, United States; ^3^E.N.T., Translational Neurosciences, Faculty of Medicine and Health Sciences, University of Antwerp, Antwerp, Belgium

**Keywords:** Mal de debarquement syndrome, vestibular diseases, pathophysiology, neuroimaging, Mal de debarquement

## Abstract

Mal de debarquement syndrome (MdDS) is a neurological condition typically characterized by a sensation of motion, which in most cases manifests after disembarking from a vehicle (e.g., boat, plane, and car). However, the same symptoms can also occur spontaneously. Two main theories of the pathophysiology of MdDS are briefly summarized here. In this perspective, we aimed to report the most recent findings on neuroimaging studies related to MdDS, as well as to suggest further potential research questions that could be addressed with the use of neuroimaging techniques. A detailed analysis of previous work on MdDS has led to five main research questions that could be addressed in new neuroimaging studies. Furthermore, in this perspective, we propose new stepping-stones to addressing critical research questions related to MdDS and its pathophysiology. We propose considerations for new studies, as well as a detailed analysis of the current limitations and challenges present when studying MdDS patients. We hope that our examination of the nuances of MdDS as a neurological disorder will contribute to more directed research on this topic.

## Overview

Mal de debarquement syndrome (MdDS) is characterized by a persistent subjective perception of self-motion (i.e., bobbing, swaying, and/or rocking), accompanied by symptoms, such as heightened visual sensitivity and anxiety. MdDS predominantly occurs in adult women between the ages of 30 and 65 years (1–5). In most cases, MdDS occurs after exposure to passive motion [for reviews, see Ref. (3, 4)]; this type of onset has been referred to as motion-triggered (MT) MdDS. MT MdDS is considered to be a disorder of poor adaptation to stable conditions after exposure to passive motion (e.g., a boat). Spontaneous-onset MdDS has also been reported (2), in which symptoms are remarkably similar to MT MdDS but occur without a motion trigger (6, 7). Re-entry of MdDS into the modern scientific literature occurred in 1987 (6), reflecting the limited duration of awareness of MdDS (4). However, recent years have seen an increase in awareness and scientific attempts to unravel the clinical and pathophysiological aspects of MdDS (3, 4).

In this Perspective, a brief recap of the current imaging literature on MT MdDS is provided along with current theories on MdDS. Although different avenues can be taken to elucidate the mechanisms of MdDS, our focus will be on how to address remaining questions regarding MdDS pathophysiology and associated features with neuroimaging and neuromodulation.

## Theory 1

A hypothesis on MdDS that was developed through neuroimaging and neuromodulation studies on human subjects contends that MdDS is a disorder of abnormal functional connectivity driven by a central neural oscillator that becomes entrained during periodic motion exposure. This central oscillator drives widespread cerebral connectivity and can toggle between high and low states. This can explain why MdDS involves motion perceptions at rest with symptoms that toggle between high and low states that can also shut off like a “light switch,” as some individuals with MdDS report. Functional connectivity reductions in MdDS subjects who have responded favorably to neuromodulation indicate that MdDS may be a disorder of over-synchronization of brain networks. This over-synchronization may have been driven by entrainment to background low-amplitude oscillating environments, such as experienced on water (3). The substrate of the entrainment process has been proposed to be the entorhinal cortex (EC), a central hub of spatial information processing located in the medial temporal lobe which has been shown to exhibit entrainability, toggles between high and low states, and is positioned to drive large-scale neural networks through its extensive connectivity. The left EC has been shown to be hypermetabolic (increased glucose uptake in FDG PET) in MdDS suffers (8). MdDS has thus been proposed as a condition of abnormally high long-range resting-state functional connectivity that is driven by this central oscillator. Specifically, this high resting-state functional connectivity occurs in sensory-processing areas, with associated MdDS symptoms attributed to the inability to desynchronize brain networks that have become abnormally yoked (3).

Over the last decade, neuroimaging studies that have included fMRI and EEG have attempted to unravel the underlying neural basis of MdDS (8–10). MdDS patients exhibit alterations in gray matter volume in visual-vestibular processing areas (e.g., V5/MT), in the default mode network (e.g., the cingulate cortex), in the somatosensory network (e.g., the postcentral gyrus), and in the central executive network [dorsolateral prefrontal cortex (DLPFC)] (9). Resting-state fMRI (rsfMRI) studies have shown an increased functional connectivity between the left EC/amygdala and visual and vestibular processing areas in the setting of decreased connectivity in multiple prefrontal areas (8). High-density EEG studies have shown that individuals with MdDS have higher neural synchronicity (over-synchronization) when they are in a higher symptom state than when they are in a lower symptom state, specifically between sensory-processing areas in the posterior parietal and occipital cortices with an important connection to the prefrontal cortex (10). For more details, please refer to Ref. (4, 5).

Limbic abnormalities in the EC have a good theoretical basis to be related to abnormal motion perception (8). The EC plays a key role in mapping one’s spatial environment (11). Furthermore, EC neurons play a pivotal role in keeping the hippocampus active during sleep, and thus in memory consolidation (12). The latter might explain why in some individuals MdDS symptoms occur only after a night’s sleep and not immediately after disembarking (3).

Neuroimaging studies have led to the implementation of non-invasive brain stimulation as a therapeutic strategy for MdDS (10). Specifically, repetitive transcranial magnetic stimulation (rTMS) over the left prefrontal cortex (8–10), an area shown to be hypometabolic in MdDS patients, has been shown to be beneficial, particularly in those with enhanced baseline functional connectivity (13, 14). It was postulated that this reduction of subjective motion is altered by the functional connectivity of the prefrontal cortex to the EC and posterior parietal lobule. The posterior parietal lobules project spatial information to the DLPFC, a pathway important in cognitive control over spatial information processing and spatial working memory (15, 16). rTMS over the DLPFC might influence multiple interconnected networks with (17) influences on mood, cognition, and visuo-spatial processing. The involvement of these other domains on the experience of MdDS was quite relevant in the choice of DLPFC as an initial non-invasive brain stimulation target (18). For a full overview, readers are referred to Ref. (4, 5).

## Theory 2

Another theory for MdDS has been formulated by Dai and colleagues (19, 20) through their animal research in subhuman primates (21). This theory suggests that MdDS results from maladaptative coupling of multiplanar information of the vestibulo-ocular reflex (VOR). The VOR ensures gaze stabilization during rotation of the head around three axes (i.e., yaw, pitch, and roll). Each of these VOR components is subject to contextually dependent adaptation. VOR adaptation can occur across different axes (22). This contextual VOR adaptation may be long lasting (23) and is the basis for suggesting that VOR maladaptation as an underlying mechanism in MdDS. A cross-axis-coupled stimulus can alter velocity storage of the VOR to produce persistent, abnormal eye movements, which were defined as vertical nystagmus occurring after horizontal rotation (20). However, it should be noted that “perverted nystagmus” (nystagmus beating in a different plane than that of the rotation) has also been noted in individuals with migraine or as an effect of medication and is thus not specific to individuals with MdDS (24, 25). This theory hypothesized that MdDS patients are failing to readjust to this new stable context due to the information retained by the velocity storage mechanism (26, 27). Based on this theory, a treatment scheme involving the recalibration of the VOR by passively and periodically tilting the head while exposing the individual to optokinetic stimulation has been developed (19). An improvement in 50% of the patients was reported following a mean of 1-year follow-up (19, 20). For a more detailed description of VOR maladaptation and the treatment protocol, readers are referred to Ref. (19–21).

Ultimately, the two hypotheses presented may not be mutually exclusive. It is possible that if VOR coupling was a brainstem manifestation of MdDS, a cortical manifestation may be enhanced functional connectivity. This remains to be empirically shown, however. The current literature, which describes the clinical profile, potential treatments and initial results from imaging studies on MdDS, has raised several key questions that could be answered by future neuroimaging investigations. As such, we have formulated four main questions for future neuroimaging studies in MdDS.

• *Question 1*: “*What brain alterations are associated with MdDS and what is the specific role of the EC/amygdala complex in motion perception and adaptation?”*
  Future studies may investigate whether metabolism in these limbic regions change as a function of symptom change.
• *Question 2*: “*Can the VOR maladaptation theory induce neural changes (by affecting the velocity storage mechanism), which may be detectable by neuroimaging?”*
  Future rsfMRI may be extended to assess whether there are cortical manifestations of successful treatment with the VOR decoupling protocol. Altered functional connectivity may potentially be related to the changes of the velocity storage since it is known that the velocity storage mechanism is modulated by the vestibulo–cerebellum interaction, specifically by the nodulus and the ventral uvula (27). As a result, lesions of these structures result in an impaired ability to realign the eye velocity vector toward the gravito-inertial acceleration vector. Cerebellar influences on cerebral resting-state networks may be thus be potentially impacted by vestibulo–cerebellar pathways.
• *Question 3*: “*What are the risk factors for developing MdDS?”*
  It is known from previous studies that MdDS affects more women than men (1). As such, age and hormonal status should be taken into account and considered as potential risk factors for developing MdDS. We hypothesize that these factors may be more determinative in the development of MdDS than the actual conditions of the motion exposure (3). This is supported by the fact that temporary land sickness is a common phenomenon in both men and women, while persistent MdDS is more common in women. Thus, individual variables that prevents the adaptation to motion from readapting, through a new adaptive process, when returning to stable conditions may be more relevant to the development of MdDS than the motion conditions themselves (3). Future PET studies could focus on hormonal receptors, e.g., estrogen receptors (28), in order to assess potential alterations in MdDS patients.
• *Question 4*: “*How are symptom variations over time and therapeutic effects related to the brain alterations seen in MdDS patients?”*
  A previous study has investigated functional connectivity measures that were related to changes in symptoms (10, 13, 14). By combining high-density EEG and fMRI, it has been revealed that specific regions in the posterior parietal and occipital cortices exhibited coupled with the changes in MdDS symptoms (10). More recently, it has been shown that improvements in MdDS symptoms, treated with rTMS over the bilateral DLPFC, correlated most strongly with a posttreatment reduction in functional connectivity between the left EC and precuneus, the right inferior parietal lobule, and the contralateral EC, part of the posterior default mode network (13). Additionally, individuals with MdDS report symptom fluctuations throughout the day, as well as improvement when being re-exposed to passive motion (2). Future studies might aim to further focus on this specific aspect, perhaps with real-time brain connectivity monitoring, since it can provide more insight into intersubject variability in MdDS symptoms and in treatment response.
• *Question 5*: “*What is the role of the associated features and comorbidities typically seen in MdDS?”*

Mal de debarquement syndrome patients tend to develop kinesiophobia (i.e., the fear of movement) and fatigue (29). Therefore, these aspects might be confounding variables in neuroimaging studies and should be taken into account. In addition, individuals with MdDS have high comorbidities with migraine, increased visual sensitivity, and mood disorders, e.g., depression and anxiety (1, 2). The association with stress should also be further investigated, since it is known that stress can exacerbate MdDS symptoms (18). Defining the neuroendocrine profile of MdDS patients (e.g., through cortisol assessment) is a potential non-invasive method to investigate any aberrant stress responses in MdDS patients. We hypothesize that MdDS patients may have an aberrant stress system, which is particularly important since neuroendocrine parameters, such as cortisol, have been associated with functional connectivity (30).

## Current Challenges and Potential Solutions

Although existing and developing imaging modalities hold great promise for the study of MdDS, progress can be impeded by particular challenges posed when studying these individuals. By summarizing these, we aim to enhance and guide future neuroimaging efforts to disentangle the pathophysiology of MdDS.

### Magnetic Vestibular Stimulation (MVS)

An important aspect to mention when considering neuroimaging studies on vestibular patients is the MVS phenomenon. It is well known that strong magnetic fields like the one used during magnetic resonance imaging studies are able to induce vertigo sensations in humans (31). MVS occurs due to Lorentz forces. This results from the interaction between the magnetic field itself and the natural occurring ionic currents in the labyrhthine endolymph fluid. A recent study showed that the endolymph could potentially induce nystagmus as a result of the MVS, as it delivers ionic current and fluid pressure, affecting the cupula (31). The trend to use stronger magnetic fields may worsen the effect of stronger Lorentz forces. Neuroimaging studies performed on MdDS patients have not reported MVS among the subjects tested, however. Eye tracking with an external camera was performed during an fMRI study at 3T (8) but no nystagmus was seen or reported. Whether nystagmus could have been present with eyes open in the dark, monitored with a scleral coil is possible, but this remains a theoretical issue. Data on over-synchronization as a function of MdDS symptoms has mostly been based on EEG, however, which has been consistent with fMRI indicating that MVS is not the major driver of fMRI findings in MdDS (10, 13, 14).

### Sample Size

Since MdDS is considered to be an uncommon disorder, acquiring sufficient sample sizes can be difficult. Therefore, future research would benefit from multi-center studies. Collaboration could enhance recruitment, e.g., through the MdDS Balance Disorder Foundation (MdDS Foundation; www.mddsfoundation.org) or through the development of an MdDS Consortium.

### Intersubject Variability

There is high intersubject variability in symptoms of MdDS patients. Therefore, the implementation of diagnostic guidelines [e.g., proposed in Ref. (4)] could help in this matter. The typical long duration between the start of symptoms and the actual diagnosis (and thus, the inclusion in scientific studies) might also give rise to several secondary and confounding factors, e.g., the development of phobic behavior, anxiety, and depression. This is a challenge in the study of any chronic disorder. However, with diagnostic criteria currently being developed and a rise in MdDS awareness, improvement in timeliness and accuracy of diagnosis is anticipated. In addition, MdDS symptoms and severity, e.g., by means of the 10-point MdDS Balance Rating Scale (32) would help to standardize future interventions.

Longitudinal studies could help model MdDS symptom variation over time, as well as the direction of brain alterations varying with symptoms and to quantify therapeutic effects. With regards to this, it has recently been shown that a positive response to rTMS treatment correlates with higher baseline functional connectivity between the DLPFC and the EC (13). As such, baseline prefrontal-limbic functional connectivity could serve as a predictor of treatment response to DLPFC rTMS stimulation in MdDS as well as a dynamic biomarker of symptom status (13).

### Case–Control Matching

Ideally, a patient and control group should be matched for as many variables as possible. Age, gender, and handedness should be considered the absolute minimum. However, for MdDS specifically, depression and anxiety levels should also be matched [e.g., by means of the Hospital Anxiety and Depression Scale, HADS (33); or the Beck Depression Inventory-II and Beck Anxiety Inventory, respectively BDI-II (34) and BAI (35)], as it is known that here is a high depression/anxiety comorbidity in MdDS patients (1, 2). In addition, the Panic Disorder Severity Scale (36) could be implemented. Furthermore, fatigue should be matched for when possible, as previous studies have already shown that chronic illnesses and MdDS specifically can lead to increased levels of fatigue (29). This could be done, for example with the Fatigue subscale of the Functional Assessment of Chronic Illness Therapy questionnaire (37).

In addition, the level of physical activity should be matched in both groups. The latter is often overlooked, although it is known that MdDS patients, and neurological and vestibular patients in general, tend to develop kinesiophobia (29). As such, this might induce non-disease specific differences in structural and functional properties of the brain. This is especially true for MdDS patients, as there is often a long time interval between symptom onset and diagnosis (38). A possible tool to quantify activity level would be the Global Physical Activity Questionnaire (39) and the Tampa Scale for Kinesiophobia (40).

### Comorbidities

The relationship between migraine and high visual dependency should be elucidated, as well as potential overlap in pathophysiology, which has already been suggested (41). Prospective studies, including MdDS (+) migraine and MdDS (−) migraine patients could develop a first insight into this. As for increased visual dependency, quantifying visual reliance, e.g., by means of measuring the subjective visual vertical or Visual Vertigo Analogue Scale could help evaluate the degree of visual dependency in MdDS patients; the association of these results with brain alterations could be made before and after treatment. Persistent postural-perceptual dizziness (PPPD) (42) is not necessarily a comorbidity, but a differential diagnosis to MdDS. Although both entities share an overlap in similar features, they differ in one important feature: symptoms during re-exposure to motion. While MdDS patients experience symptom alleviation when re-exposed to motion (2), this can worsen symptoms for PPPD patients (42). As such, this element might be employed to distinguish both pathologies. In addition, this could help discriminate MdDS from other vestibular disorders and motion sickness (43).

### Risk Factors

In addition, risk factors for developing MdDS, such as age and hormonal status, need to be further elucidated. It has been shown that the cholinergic and serotonergic systems are biological mediators of hormonal influences on the brain (44). Furthermore, menopausal hormone changes can modulate neuronal activity and as such, contribute to age-related memory loss and the development of neuropsychological disorders (44). Therefore, future studies might aim to focus on investigating these neurotransmitters by means of PET. In addition, the hormonal parameters of MdDS should be investigated. Whether hormonal levels correlate with brain alterations found in MdDS patients and, perhaps more practically, whether further treatment should consider these hormonal fluctuations on therapeutic choices remains to be seen.

## Conclusion

Despite considerable challenges, neuroimaging offers the prospect of a greater understanding of MdDS as a central nervous system disorder, as well as a platform for the further extension and improvement of therapeutic strategies to treat an otherwise intractable disorder. MdDS itself may be uncommon, but the relevance of understanding MdDS to other disorders of abnormal entrainment is very high. The complexity of sensory stimuli in our environment and in particular the greater role that transportation plays in our daily lives, will make understanding the pathophysiology of MdDS of increasing relevance.

## Author Contributions

AO and VM: conception and design, interpretation of data, drafting the submitted material, and critical review. FW and Y-HC: conception and design, interpretation of data, critical revision, and supervision.

## Conflict of Interest Statement

The authors declare that the research was conducted in the absence of any commercial or financial relationships that could be construed as a potential conflict of interest.
